# Supplementary material for: Perceiving fingerspelling via point-light displays: The stimulus and the perceiver both matter
Source: PLoS One. 2022 Aug 16;17(8):e0272838. doi: 10.1371/journal.pone.0272838 (PMC9380947; doi:10.1371/journal.pone.0272838)
Supplement: Attachment — Submitted filename: Response to reviewers 2.pdf [file pone.0272838.s002.pdf]

**Response to Reviewers**
Leannah, Willis, & Quandt - 2022

***Editor's Comments***
*Thank you for submitting your manuscript to PLOS ONE. After careful consideration, we feel that it has merit but does not fully meet PLOS ONE's publication criteria as it currently stands. Therefore, we invite you to submit a revised version of the manuscript that addresses the points raised during the review process.Please address the points raised by R2, considering my comments below.*

*In principle I agree with R2's encouragement to perform a linear mixed effects analysis. LME has significant advantages over ANOVA, many of which are relevant to your study (e.g., heterogeneity of variance; item effects; ability to include multiple covariates like word length and age; and the ability to include variable-by-subjects random slopes (e.g., distinguish individual variability in real/nonreal differences from group means) to better account for individual variability). That said, you clearly preregistered a specific set of analyses involving ANOVAs and t-tests so I feel it is hard to make acceptance of your paper contingent on reporting such an analysis - since this would be fairly redundant with what you already present (albeit perhaps slightly more rigorous, and potentially more sensitive). Furthermore, although in principle I believe that LME analyses are preferable to ANOVA in virtually any repeated-measures situation, I don't see that R2 has provided a particularly compelling argument as to why your present analyses are flawed or insufficient for testing your hypotheses, and as such I feel that your pre-registered approach satisfies PLOS ONE's acceptance criteria. I would nonetheless strongly encourage you to become proficient with LME and use it in future analyses, because it is increasingly expected.*

*Nonetheless, I would ask you to revise the results in a different way. Specifically, as currently presented the analyses you report in the Results section under Planned Analyses do not follow the analyses that you describe in the Methods section under Data Analysis - Planned. Your methods describe ANOVAs first and then t-tests, but your Results start with the t-tests. Besides the confusion arising from such inconsistency, one typically expects a multi-way ANOVA to be presented first, followed by t-tests that further clarify simpler contrasts encompassed in the ANOVA. At present, for example, although you report the significant effects from your ANOVAs, you do not clarify the direction of such effects. I do also encourage you to address R2's question as to why you report both a main effect of Word Type and a t-test of the same contrast.*

Thank you for these comments; we appreciate the feedback. A separate ongoing research study from our lab, also using large-scale online data collection, is planned to use an LME analysis. We agree with you that this method is becoming more common and as such we are becoming more familiar with those approaches as well.

We have fixed the order of the Analyses in the Results section, so they should now have a more logical flow as you suggested. We also have clarified the direction of all significant effects. We also addressed the issue with presenting the Word Type information in two ways--this was an error and we have fixed it. Thank you and Reviewer 2 for noticing it.

The text on pages 13-15 has been extensively added to and re-ordered to accommodate those suggested changes.

*I observe that R2's suggestion of a scatterplot version of Figure 4 with only the Deaf participants, could be achieved simply by color-coding the data points according to group, as you have used color in other scatterplots.*

Based on this suggestion from both the Editor and Reviewer 2, we made significant changes to that Figure (now, Fig 5 due to re-ordering). We appreciate the suggestion and agree the new figure is more informative and helpful to readers.

*Please be sure that your repository on [osf.io](osf.io) is not private, as we cannot move forward with publication if open access to materials is claimed but not actually provided.*

We have made the OSF repository public and it should be viewable to anyone now.

**Reviewers' comments:**

*Reviewer #1: I want to clarify one of my comments from the original review. This was more a comment as something to consider for the future and not something which has direct bearing on the present manuscript.*

*In my original review, I wrote, "It may be instructive to replicate the present study using stimuli from signers with different linguistic backgrounds."*

*The response to this was, "We would like to clarify that this study included signers from across a wide variety of backgrounds…" however the authors seem to be referring to the background of the *study participants* rather than the person(s) from whom stimuli were created.*

*What I'm suggesting -- again, not something to undertake for the present study -- is creating *stimuli* from signers of varying backgrounds because impressionistically, fingerspelling is easier to comprehend from native signers. But, as this is the minority of the signing community, it would be informative to know how degradation of signal from non-native signers is perceived.*

We thank the reviewer for explaining this point again. We did misunderstand the comment the first time, but now we fully understand and completely agree that it would be interesting to investigate how non-native signers produce fingerspelling differently, and how those productions would be viewed and comprehended. Thank you for sharing your idea.

*Reviewer #2: The authors have made thoughtful responses to the comments and have addressed many of the issues raised. This paper makes an important contribution to our understanding of fingerspelling perception with a robust dataset, including perceiver characteristics (age, AoA, hearing status, fluency) and stimuli characteristics (high/low, and real/fake). My comments on this revision are specific to a few remaining points regarding the statistical analysis:*

*p. 12. I appreciate the nuance in choosing statistical analyses, but I will push back on one point. I would encourage the authors to analyze the current dataset using a mixed-effects regression model where you can look at multiple predictors of accuracy: length of the word (not currently analyzed), type of word (real/fake), population (deaf/hearing), and number of markers (high/low). This would also allow you to include random effects of participants and items. I do not see a compelling reason not to do a mixed-effects model in favor of an ANOVA. There were 27 items across over 260 participants—with this robust dataset, a mixed-effects regression would provide a more sensitive analysis that includes item-level effects.*

We understand the reviewer's explanation, and we appreciate their comments. We note that the editor has weighed in on this particular topic, and so we will continue ahead under their guidance. Thank you sincerely, and you will see in our response to the editor that while we believe it is best to stick with the planned analyses for this paper, we are indeed moving our data analysis methods towards LME for future studies.

*p. 12: I am not clear on the distinction made between the ANOVA that included Word Type as a fixed-effect, with the paired samples t-test comparing the effects of Word Type on accuracy. What was paired in this test—was this at the individual, i.e. responses from each participant were compared for real vs fake words? Please clarify.*

In the course of clarifying our results section and standardizing it, we no longer have a separate paired t-test to examine this effect. All Word Type, Number, and Group effects are analyzed solely in the ANOVAs now.

*p. 15. I appreciate the addition of the post-hoc test that probes the interaction effects between realness and number on confidence. I don't see this reported in the test. I suggest adding the statistical results of the post-hoc test (i.e. t-test and p-value) to the text.*

We have gone through the text to ensure that post-hoc test results have been added to the results wherever relevant. The text on pages 13-15 has been substantially revised.

*p. 15-16: When reporting the interaction between Word Type and Age, I recommend including the t-test results here as well.*

The analysis that is referred to here uses Age (at the time of the experiment) as a covariate. We do currently include the F test which reveals the significant interaction between Word Type and Age. We are not sure what additional t-test would shed more light on this significant effect.

*Figure 4: The relationship between AoA and Accuracy/Confidence in figure 4 is really striking. I would love to see how this scatterplot looks for just the deaf participants. Since you have such an impressively large sample of deaf participants, you have a unique opportunity to show how AoA among deaf signers affects an area of sign language perception not previously explored, namely degraded fingerspelling perception.*

We have now created a new Figure (now #5), in which Deaf and Hearing groups are shown with different colors so it is possible to see how the effects break down by hearing status. We agree this is a better use of our large dataset and shows some unique information.

Minor note:

*p. 5: Lines 85 and 90 both mention the ability to perceive speech “at a loud party.” I’d suggest removing one of those references.*

Thank you for catching this awkward phrasing. We have edited this paragraph to be more clear and less redundant.

*I am unable to access the data on OSF—it is noted as restricted access.*

We have changed the settings to public.
