# Supplementary material for: Perceiving fingerspelling via point-light displays: The stimulus and the perceiver both matter
Source: PLoS One. 2022 Aug 16;17(8):e0272838. doi: 10.1371/journal.pone.0272838 (PMC9380947; doi:10.1371/journal.pone.0272838)
Supplement: Attachment — Submitted filename: Response_to_reviews.docx [file pone.0272838.s001.docx]

Leannah, Willis, & Quandt

Response to Reviewers

**Additions to the manuscript text are shown in bold text.**

*Reviewer #1: This clearly and succinctly written article presents a noel study of perception of degraded fingerspelling. This line of inquiry is related to previous research on speech intelligibility when speech sounds are degraded, such as at a party when there is significant noise interfering with the linguistic signal. This is a large data set allowing for many exploratory analyses which could provide the foundation for a variety of future lines of work, especially because the stimuli are openly shared by the authors.*

*Several comments to consider:*

*1. It may be instructive to replicate the present study using stimuli from signers with different linguistic backgrounds. The present study uses deaf native signers, and while this is often used as the gold standard, deaf native signers make up a small percentage of the deaf ASL-using community. Thus, wouldn’t it do more to replicate real-world experiences to have data from signers with different backgrounds which are more representative of the deaf community at large? A guiding question would be, “to what extend does the language background of the signer affect fingerspelling comprehension.”*

We thank this reviewer for their interest and enthusiasm about our paper. We would like to clarify that this study included signers from across a wide variety of backgrounds--both hearing and deaf, including those who learned ASL from an early age (i.e., native signers) and those who first acquired ASL as an adult. This information is included in the manuscript on page 7, Table 2, and throughout the results and discussion sections in which we compared Deaf and Hearing groups and conducted analyses across different fluency levels and ages of ASL acquisition.

We also added text on page 9 to more clearly state the following: “**All participants were required to know some ASL, but the sample included signers across a wide range of fluencies—from deaf native signers through to adult beginner ASL students.**”

*2. In Lines 404-405, you have the parenthetical, “cites from general visual perception field.” This looks like a note-t0-self. Perhaps the intention was to put references related to this field?*

Thanks to this reviewer for pointing out this error. We have filled this section in with the relevant citations.

*3. Another potential future line of inquiry: to what extent do confidence ratings correlate with other measures of ASL fluency, such as the commonly used ASLPI? Given that fingerspelling is considered a difficult skill, especially for L2 learners, it would be interesting to know whether such a correlation exists.*

We agree that it would be very interesting to examine how accuracy or confidence on this task correlates with ASLPI or ASL-SRT scores. While we don’t know the answer to that question, we hope that by making the stimuli available to other researchers, other researchers can use the stimuli to explore new lines of inquiry.

***Reviewer #2:*** *This study uses a point light display (PLD) presentation of fingerspelled place names (real and fake) to investigate deaf and hearing ASL signers’ accuracy and confidence in perceiving fingerspelling. The authors compared two groups of signers (hearing and deaf) on their performance in two types of degraded stimuli (high markers vs low markers). They found that deaf signers performed better overall, that highly marked stimuli were easier to perceive than lower marked stimuli, and that real names were more accurately perceived than pseudowords. In addition, exploratory analyses on chronological age and hearing status among early-exposed signers showed that both factors had a significant effect on accuracy, at least for some stimuli.*

*The strengths of this paper are that it adds to our current limited understanding of how signers perceive fingerspelling, and specifically how a specific type of reduction in the signal (replacing full hands and bodies with point light displays) impacts perception. The sample is large (over 260 participants, including 108 deaf participants). The study predictions were pre-registered and all of the data is publicly available, including the PLD videos, which were previously created by one of the authors (although the project does not appear to be currently accessible on the OSF site). My suggestions for the paper largely center around improving the motivation and rationale for both the study itself, the way the high vs low stimuli were created, and the comparisons among deaf and hearing signing groups. Particularly for readers of PLOS One who may not have extensive prior knowledge of fingerspelling and how it is perceived, a more nuanced introduction would be helpful.*

We have added the data to the OSF repository and it is now available to be viewed. We have also made changes to the introduction in response to several comments from both reviewers, so we hope this has provided the details and nuance this reviewer hoped for.

*1. The pre-registered predictions all make sense (deaf > hearing, high > low, etc.). I was wondering if you also had some overall predictions about the accuracy of fingerspelling perception overall. That is, among the most experienced signers (deaf signers with early ASL exposure), what is the overall prediction regarding how easy or hard it would be to identify fingerspelling from PLD stimuli?*

We did not formally predict the difficulty of understanding the PLDs, partly because there was no prior work in this area and the fact that we use a novel “accuracy” rating system. However, all research team members are ASL signers, with a range of fluencies among us. Anecdotally, we noticed that Deaf native signers seemed to have no trouble understanding the real place names when shown with the high number of markers. In contrast, one hearing adult-learner of ASL had much more difficulty recognizing any stimuli from PLDs alone. So from our lived experience, we did think that the most proficient signers would easily recognize the PLDs quite well.

*2. p. 4: In the introduction, authors note that fluent signers fingerspell very fast. It also seems relevant that in addition to speed, many fingerspelled signs become somewhat reduced such that they are producing more of a holistic shape than each individual letter (maybe not to the extent of a lexicalized fingerspelling, but, e.g. in how they produce double letters).*

This is a good point. We have added the following on page 4: “**A fluent fingerspeller may also blend adjacent letters together, resulting in more efficient movements.**”

*3. p. 4: I would encourage caution in referring to fingerspelling as a method of teaching deaf children how to read and write, since the mechanism and extent to which this works is a matter of debate, so without context a reader unfamiliar with fingerspelling could use some context here. Also—I’m not sure if the notion of lexidactylophobia is relevant to this paper.*

We have removed some of the text about this issue on page 4, deeming it to not be closely related to the goals of our introduction. On pages 4-5 we now have the following text: “More recent work suggests that fingerspelling **may provide** a visual and linguistic link to the English language, **which may help** deaf signers acquire English vocabulary and syntax (Stone et al., 2015; Grushkin, 1998; Wilcox, 1992).

*4. It wasn’t immediately clear if the experiment was conducted remotely/online—could you clarify?*

We have added another mention of this on page 9: “**The entire study was conducted remotely via the internet.**”

*5. Participants were grouped according to hearing status (deaf vs hearing), which is presumably a proxy for knowledge of ASL. But then later analyses add age of acquisition and ASL fluency as specific grouping variables. This is handled somewhat in the Discussion, however I’d like to see some rationale at the start of the study for hearing status as a grouping variable.*

We wanted to examine fingerspelling perception both as a function of hearing status, and as a function of ASL knowledge (measured by fluency and age of acquisition). We have added the following on page 7 of the Introduction: “**It is possible that the physical state of being deaf may change people’s perceptual capacities, and it is also possible that differences in fingerspelling perception are more closely tied to overall ASL fluency. Analyzing the data in multiple ways (e.g., looking both at hearing status and at fluency) allows us to better understand the effects of hearing status, language experience, and fluency—as they relate to signers’ perception of fingerspelling.”**

*When age of ASL exposure was added, I was also wondering if the hearing participants provided information about where they learned ASL—were these individuals with deaf parents or family members?*

The hearing signers in this study ranged widely from children of deaf adults (CODAs who learned ASL from birth) to adult ASL learners with only introductory knowledge of ASL. Table 2 shows that the mean age of ASL acquisition for the Hearing group was 16.4 years, with a standard deviation of 7.8.

*6. Similarly, it would be good to include some more context for the prediction about interpreting real vs pseusoword place names. This is related to the discussion of holistic perception. If signers are accustomed to comprehending fingerspelling holistically, then they might approach the task by bringing their previous knowledge into play. However pseudowords must be processed as individual letters as there is no target words on which to map the fingerspelled pattern. In the current task, signers didn’t know ahead of time whether a given stimuli is going to be a real or fake place name, so they likely had to use a flexible strategy to first perceive the letters, and then determine whether the letters mapped closely onto a known place name. I suggest some additional discussion about how fingerspelled is processed as it unfolds that might help motivate the comparison of real and pseudowords.*

We touch on this issue at several points throughout the manuscript, as this does indeed relate to our original motivations for comparing both real and pseudo place names. Our analysis is not sensitive to the time-related processing which unfolds during the perception of fingerspelling, hence why we don’t emphasize this point in the paper. However, the reviewer makes a good point and future work using eye-tracking or psychophysiological methods which can analyze perception on a short time scale would be interesting to conduct.

*7. Methods:*

*-were participants informed that some of the items would be real and some would be fake?*

Yes, they were informed of this. We have added to page 11: “**First, participants were instructed that they would see videos showing a person signing location names, which could be real or made up (fake). They were also told they would have only one chance to view each video. Participants completed two practice trials before starting the task, which allowed them to become familiar with the task.**”

*-did you control how closely the pseudowords resembled real place names? Some seem to be combos of two real words (“fadestring”) while others have little resemblance to a real word (“unteria”), while “hillopolis” is somewhere in between. What were the parameters for choosing the pseudowords, and did you consider how specific features would influence performance?*

We did not control for this, although we see why that would be wise for future studies. When we created these stimuli several years ago, we had not planned this exact study yet. We selected “nonsense words” from [www.soybomb.com](http://www.soybomb.com), which we subjectively deemed could be plausible place names, as we explain on page 8. The nonsense word generator uses large sets of real English words to create new pseudowords that reflect the frequency of various phonemes in the English language. In the future, researchers could use similar methods to examine specific linguistic properties of novel fingerspelling stimuli presented via PLDs, which may lead to some interesting findings.

*8. Results:*

*Can you clarify how accuracy was calculated? Mean accuracy is reported as 2.9 for the deaf and 1.9 for the hearing signers—is this the mean accuracy on an individual item? If so, can you explain the reason for calculating accuracy at the mean item level, rather than an overall accuracy score out of 4*27? It seems that you would get more robust information from overall accuracy, which can range from 0 to 108, than from an average of averages which can only range from 0 to 4. On the other hand—providing a mean of each individual item provides more data points, so there is a trade-off in the number of data points vs the range of possible scores.*

Yes, the reviewer’s understanding is correct--these values of 1.9 or 2.8 are average values across all items. We did not sum all of their scores because that would prevent us from looking at the item-level analyses. For instance, the accuracy scores on Real names with a Low Number of Markers items must be represented by the item-level accuracy value (0-4). For the sake of consistency across different analyses, we use averaged item-level accuracy throughout the paper. While the absolute range of possible scores is smaller than it would be if we summed the scores, the differences are still measurable as shown by our analyses and data visualizations. Perhaps we misunderstood the reviewer’s point--if our response does not clear this up, we would be happy to learn more about their ideas.

*Perhaps you might consider at least plotting the distribution of scores across participants—did participants tend to be internally consistent in accuracy across items, or was there wide variation?*

We have calculated the internal consistency of both Accuracy and Confidence. The following has been added to page 13:

“**We conducted a reliability analysis to evaluate the internal consistency of both Accuracy and Confidence data on participants’ responses to all four types of stimuli (Real and Pseudo x High and Low). The Cronbach’s Alpha for Accuracy across all stimului was 0.94. The Cronbach’s Alpha for Confidence across all stimului was 0.93. In other words, a participant who was highly accurate on one type of item was likely to also be accurate on the other types of stimuli.**”

*9. Also—since this is such a large dataset (over 260 participants with 27 items each), I would suggest controlling for random effects (such as participant, item, number of letters) using a mixed-effects model with accuracy and confidence as outcome.*

We know that the choice of statistical tests for a large dataset like this one can be a source of ongoing discussion, and we do hope the reviewer sees the effort we put into characterizing the data clearly and from several perspectives. For instance, we included analyses based on self-reported ASL Fluency and also on Group (Hearing and Deaf), which each allow us to draw different, but converging, conclusions about the data. We included both correlational and ANOVA analyses, which again suggested overlapping conclusions, and we have provided plots of all the ANOVA results with error bars to aid in the visualization of the effects we found. Given the pre-registered hypotheses comparing between groups and fluency levels, and comparing between different types of signers, we have conducted our study in a way that directly tests those particular comparisons.

*10. I am not sure I understand the motivation for the exploratory analysis that uses chronological age as a covariate, since these are all adult participants. Is there previous evidence that people improve in their ability to perceive degraded linguistic information over time? Is this intended to measure effects of ASL experience? If so, then why not use years of ASL experience as the co-variate instead?*

In the section named “Data analysis - exploratory”, we explain that this analysis using Age as a covariate was run to see if Age was a confounding variable (see lines 251-254). We added this new text to make this point even more clear: “This analysis was planned to check for significant effects of the confounding factor of Age, **since the two groups did have different average ages. The analysis was not motivated by any *a priori* predictions about age.”**

We also added the following text on page 22 following the discussion of the Age effect: “**Given that these findings were the result of an exploratory analysis, future work should investigate this question using planned analyses targeted to this question**.”

Because our analysis did reveal a pattern involving Age, we report those findings, and then provide the post-hoc speculation about why these patterns may have emerged. In order to appropriately probe this question (e.g., “*do* *people improve in their ability to perceive degraded linguistic information over time”*?) it would be ideal to design a new experiment.

*This is only addressed in the Discussion, where knowledge of place names is used as a potential explanation for the increase in performance with age. The place names all seem fairly common for individuals even at the young end of the sample. Did you probe whether people knew the items in the real place name list after the experiment?*

We did not probe whether people knew the place names, though that would be an exciting addition to future work using these (or similar) stimuli. The place names do seem relatively standard, but perhaps in our sample (a diverse selection of signers across the country, not all affiliated with universities), younger people may be less familiar with these place names.

*11. Similarly, the exploratory analysis of the effects on hearing status among early signers only needs better motivation. What is it about hearing status did you suspect might lead to differences in accuracy or confidence—is it experience perceiving ASL, or perhaps even influence of knowledge of English?*

We have added more text to the “Data analysis -- exploratory” section, in order to help explain our motivation on page 13: “**The importance of early language exposure has been noted often in the literature [30,31]. Additionally, the independent effects of deafness and sign language experience upon perception in deaf signers are not well understood, in part because of the difficulty in experimentally separating sign language experience and the physical state of being deaf. Because we had a large and diverse dataset, we opted to examine a subset of our participants to shed light on these essential questions. Thus,** we conducted an exploratory analysis **between Deaf and Hearing signers** who acquired ASL at or before age three and rated themselves as fluent.”

*12. I would like to see some additional consideration of what the PLD stimuli can tell us about real-world fingerspelling perception. Although PLD is one type of degraded stimulus, it is quite different from say, perceiving language in (audio or visual) noise. Instead, using the PLD stimuli might provide insight into what aspects of fingerspelling signers are paying attention to—e.g. how much information do signers get from the more proximal movement patterns of the elbow and wrist vs. the more detailed movements of the finger joints?*

We have added the following on page 19 to more directly address this idea: “**The differences between the perception of stimuli with greater visual information—including more markers on the individual finger joints—suggests that while the information from these joints does contribute significantly to fingerspelling comprehension, it is possible for people to understand fingerspelling with only limited information about finger joint movements**.”

*Here it might also be helpful to discuss how you decided what markers to keep in the high informative vs low informative displays—were markers systematically manipulated, and if so, how?*

We have edited the caption for Figure 1 and made a small addition to the text on page 9 to more clearly explain that the placement of the markers for all stimuli in Low and High conditions were consistent:

Figure 1: “**These marker placements were consistent across all stimuli.**”

Page 9: “**Across all stimuli**, the remaining markers were on the fingertips and the knuckle between the proximal and middle phalange for the Low versions. We created each stimulus’s High and Low versions from the same recorded fingerspelling production, so underlying movements and timing were identical.”

*Figure 5: I suggest some label changes for clarity, so that instead of “realness” and “number,” perhaps something like “word type” and “informativity.”*

We have gone ahead with changing “Realness” to “Word Type” as suggested. We agree this is more clear. However, we felt that Number was the best descriptor for this variable, so we retained Number as a label name. We hope the reviewer will agree this is satisfactory.

*p. 18: for the interaction between realness and number on confidence, did you perform a post-hoc test to understand what factor was driving the interaction?*

Thank you for the suggestion. On page 16 we have now added the following text: “**Post-hoc tests on the significant interaction between Word Type and Age showed that for stimuli with a High Number of markers, the difference based on Word Type is greater, whereas for Low stimuli, there was a smaller difference between Real and Pseudo names.**”

*p. 18: the reference to Figure 5 should, I believe, reference Figure 6. Similarly, the references to figures 6 and 7 (p. 21) should reference Figures 7 and 8.*

Thank you--we have fixed those errors.

*p. 27—minor note, but in the future directions, a suggested is made to probe fingerspelling in deaf people who do not use sign language—is the suggestion that some deaf people learn fingerspelling in isolation but otherwise rely on spoken/written language?*

The reviewer is correct that this was an unclear statement. We have now changed the text on page 27 to read:

“Future researchers may want to recruit targeted groups of hearing native signers and **deaf people who learn to sign in adulthood** to understand better the unique effects of AoA upon fingerspelling perception.”
