# Supplementary material for: Perceiving fingerspelling via point-light displays: The stimulus and the perceiver both matter
Source: PLoS One. 2022 Aug 16;17(8):e0272838. doi: 10.1371/journal.pone.0272838 (PMC9380947; doi:10.1371/journal.pone.0272838)
Supplement: Attachment — Submitted filename: Response to reviewers_3.docx [file pone.0272838.s003.docx]

Response to reviewers:

*One note: On p. 16, the authors write: Post-hoc tests on the significant interaction between Word Type and Age showed that for stimuli with a High number of markers, the effect of Word Type is greater, whereas for Low stimuli, there was a smaller difference between Real and Pseudo names. This seems to be explaining an interaction between word type and number, not word type and age, but no word type X number interaction is reported.*

We have fixed this error—there was an erroneous copy/paste of another sentence inserted here.
